# In-Depth Evaluation of Ultrasonically Welded Al/Cu Joint: Plastic Deformation, Microstructural Evolution, and Correlation with Mechanical Properties

**DOI:** 10.3390/ma16083033

**Published:** 2023-04-12

**Authors:** Junqi Li, Johannes Zillner, Frank Balle

**Affiliations:** 1 Walter-and-Ingeborg-Herrmann Chair for Power Ultrasonics and Engineering of Functional Materials, Department of Sustainable Systems Engineering (INATECH), Faculty of Engineering, University of Freiburg, 79110 Freiburg, Germany; 2 Freiburg Materials Research Center (FMF), 79104 Freiburg, Germany; 3 Fraunhofer Institute for High-Speed Dynamics (EMI), 79104 Freiburg, Germany

**Keywords:** ultrasonic welding, dissimilar joints, plastic deformation, microstructure

## Abstract

Ultrasonic metal welding (USMW) is widely used in assembling lithium-ion (Li-ion) battery packs due to its advantages in joining dissimilar and conductive materials in the solid state. However, the welding process and mechanisms are not yet clearly understood. In this study, dissimilar joints of aluminum alloy EN AW 1050 to copper alloy EN CW 008A were welded by USMW to simulate the tab-to-bus bar interconnects for Li-ion battery assembly. Qualitative and quantitative investigations were carried out on plastic deformation, microstructural evolution, and the correlated mechanical properties. During USMW, the plastic deformation concentrated on the Al side. The thickness of Al was reduced by more than 30%; complex dynamic recrystallization and grain growth occurred near the weld interface. The mechanical performance of the Al/Cu joint was evaluated with the tensile shear test. The failure load gradually increased until a welding duration of 400 ms, and then remained almost constant. The obtained results showed that the mechanical properties were greatly influenced by plastic deformation and microstructure evolution, which provides guidance for improving the weld quality and the process in general.

## 1. Introduction

Recently, the concerns regarding climate change and energy exhaustion have led to an increasing demand for battery electric vehicles (BEVs) including electric, hybrid electric, and plug-in hybrid electric vehicles [1,2,3]. The performance of BEVs is highly dependent on the power and energy density of their battery packs. Due to their high energy density, low self-discharge, and portability, lithium-ion (Li-ion) electrochemistry-based secondary batteries are now widely used for BEVs [4,5]. Typically, an automotive battery pack is manufactured in a pack–module–cell structure and consists of several hundred to a thousand interconnected individual cells, which are connected through tab-to-tab or tab-to-bus bar joints [5,6,7]. Copper (Cu) and aluminum (Al) are commonly used as materials for battery tabs and bus bars due to their good ductility, corrosion resistance properties, and high thermal and electrical conductivities [8,9,10]. A reliable joining technique is essential to ensure a robust mechanical and electrical connection, as the failure of a single joint can result in the invalidity of the entire battery pack.

The most commonly used joining techniques are resistance spot welding (RSW), pulsed tungsten inert gas (TIG) welding, laser beam welding (LBW), and ultrasonic metal welding (USMW). Depending on the material properties and contact geometry, these welding techniques have their own advantages and disadvantages [4,11,12,13]. When compared with the rest of the welding techniques, USMW is one of the major joining techniques used for assembling battery packs due to its advantages of low energy consumption and short welding time. Additionally, USMW is more suitable for welding multiple thin sheets of dissimilar highly conductive materials, whereas several challenges, such as large differences in melting temperature and reactions between dissimilar metals, limit the conventional fusion welding techniques.

USMW is a solid-state welding process that uses ultrasonic shear vibration to produce relative motion between joining partners (typically in sheet or wire forms) clamped under pressure [8,14,15]. Generally, there are three main process parameters used to control the welding process: oscillation amplitude, welding force, and welding time/energy. The comprehensive effects of process parameters on joint quality have been investigated by a large number of researchers [16,17,18,19,20,21,22,23,24,25]. A reasonable combination of all three process parameters is critical to ensure a robust mechanical and electrical connection. Hence, the design-of-experiment (DoE), regression analysis, and machine learning techniques have been applied to determine and optimize the process parameters [16,22,23,24,25]. Such methods are commonly classified as “black box” models because the process physics and the underlying bonding mechanisms are not required to be understood [15].

To evaluate weld quality, welded joints are usually evaluated by the T-peel, U-peel, and tensile shear tests, where the failure load and failure modes are used to assess the mechanical performance of the joint [26,27,28]. Das et al. [27] categorized the weld quality into three classes: under-weld, good weld, or over-weld. While under-weld joints are characterized by interfacial separation with no or partial adhesion of materials, a partial or full circumferential fracture was observed around the perimeter of the weld nugget in the case of the over-weld. The good-weld joints show a limited interfacial separation with material tearing and had a maximal failure load in the test.

During the actual operation of a battery module, the interconnects may be damaged due to the dynamic loading, random vibration, as well the dynamic behavior of batteries during charging and discharging [29,30]. Thus, the interconnects should also be resistant to crash impact, vibration, and fatigue [5,11]. However, few studies have focused on the dynamic deformation behavior of welded joints. Lee et al. [10] proposed waveform design methods to reduce the parasitic vibration and maintain the quality of the welded joint during the welding process. Kang et al. [31] used a one-dimensional continuous vibration model to analyze the stress distribution in the battery tab. According to their results, the residual stress induced by the resonant flexural vibration can cause fatigue fracture near the weld area. Zhao et al. [32] developed a fatigue life cycle model to predict fatigue life of ultrasonically welded Al/Cu joints. Additionally, the service condition may affect the mechanical strength of the interconnects. According to [33], ultrasonically welded Al/Cu joints are reliable in working environments with temperatures below 150 °C, because no obvious intermetallic compounds (IMCs) form at the weld interface. Nevertheless, inhomogeneous thermal expansion may introduce shear stress in the weld area due to the different physical properties of the base metals [34]. Further studies on the effects of dynamic loading, thermal input, and corrosion are needed.

Several other studies have characterized the welding process via different measuring systems. The temperature evolution during the USMW process has been measured by embedding thermocouples at the interface or by observing the interface using an infrared camera [17,18,19,20,21]. The temperature at some localized spots may reach or exceed the melting point of the joining partners, even though the average temperature at the weld contact is below the melting point. High-speed camera and laser-doppler vibrometry-based devices have been applied to observe the relative motion between a sonotrode, joining partners, and an anvil [35,36,37,38]. It was indicated that the USMW process involves different welding stages such as a slip stage, slip–stick transition stage, stick stage, and over-welding stage. Such investigations can improve the understanding of the welding process. Thus far, few studies have provided insights into the bonding mechanism of Al–Cu ultrasonic welding. Yang et al. [39] reported that mechanical interlocking, localized metal melting, significant diffusion, and recrystallization can occur during USMW. However, the bonding formations and the microstructure at the weld interface vary with different material combinations and processing conditions. While Zhao et al. [17] and Wu et al. [40] determined the intermetallic compounds (IMCs) of Cu_9_Al_4_, no IMCs were observed by Balasundaram et al. [41]. Fujii et al. [42] used texture analysis to investigate the bonding mechanism and proposed that dynamic recrystallization occurred during USMW. Ma et al. [43] performed molecular dynamics (MD) simulations to analyze dynamic weld evolution, which suggested that weld formation involved several processes, including microweld formation, tear deformation and fracture of microwelds, Al surface flattening, and complete weld formation.

In view of the above, it can be found that more systematic and theoretical analyses are still required for the scientific understanding of weld formation mechanisms. Thus, the overall purpose of this work was to contribute to the explanation of USMW to improve welding quality. For this purpose, the plastic deformation of the joining partners and the microstructure development at the weld interface were qualitatively and quantitatively investigated. Additionally, their influence on the joint strength was discussed.

## 2. Materials and Methods

In this study, commercially pure 1 mm thick sheets of aluminum alloy EN AW 1050 (Al) and copper alloy EN CW 008A (Cu) both in half-hard condition were used to simulate the joining of battery tabs and bus bars. The selected mechanical properties of the base materials are listed in Table 1.

**Figure 1:**
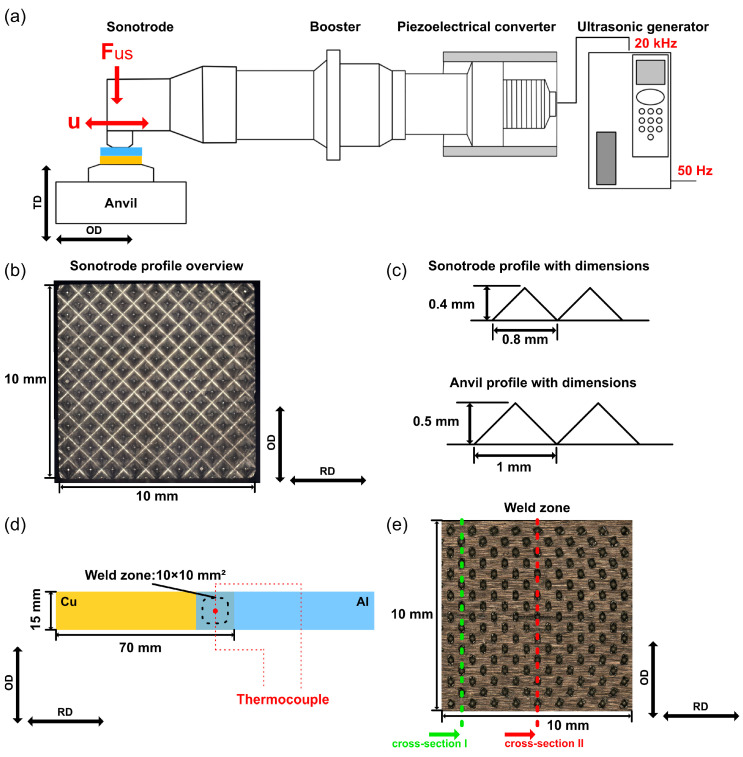
Experimental setup: (**a**) schematic of an ultrasonic welding system, (**b**) optical micrographs of sonotrode profile, (**c**) dimensions of sonotrode and anvil profiles, (**d**) configuration of the joining partners, and (**e**) cut positions of cross-sections.

A Herrmann HiS VARIO B (Herrmann Ultraschalltechnik GmbH & Co. KG, Karlsbad, Germany) welding system with a working frequency of 20 kHz was employed for this study. Figure 1a schematically depicts the main components of the ultrasonic welding system. The ultrasonic generator converted the mains AC voltage with a frequency of 50 Hz to a high-frequency voltage of 20 kHz. In the piezoelectric converter, this high frequent electrical oscillation was transformed into a mechanical oscillation of the same frequency based on the inverse piezoelectric effect of the piezoelectric crystal. Between the converter and sonotrode, a booster was mounted to amplify the oscillation amplitude. This oscillation amplitude was further amplified by the sonotrode. The transverse oscillations from the sonotrode introduced shear loads into the weld zone.

As shown in Figure 1b,c, both the sonotrode and the anvil had a pyramidal profile on the coupling surface to the joining partners, which served to prevent excessive slippage and ensure a good transmission of the oscillation. The dimension of the sonotrode surface was 10 mm × 10 mm, while the anvil side was 40 mm × 40 mm. Furthermore, the welding force (F_us_) was applied with a pneumatic cylinder, which applied constant pressure on the joining partners throughout the welding process.

Both joining partners were cut from sheet material and had dimensions of 70 mm × 15 mm × 1 mm. Figure 1d shows the configuration of the joining partners. The length of the joining partner was set to be parallel to the rolling direction (RD) of the sheet, which was perpendicular to the oscillation direction (OD). In this study, the thickness direction (TD), OD, and RD served as the specimen coordinate system. Al and Cu were placed on the anvil as the upper and lower joining partner, respectively. The Al/Cu joint had an overlap area of 15 mm × 15 mm, and the weld zone was positioned at the center of the overlapped area. The main welding parameters were selected as follows: an oscillation amplitude (u) of 28 μm, a welding force (F_us_) of 700 N, and welding time varied systematically from 75 ms to 700 ms. Before the welding process, the surfaces of both joining partners were cleaned using ethanol in an ultrasonic bath to remove the contaminants. No further surface treatment was applied to change the surface condition.

To evaluate the monotonic mechanical properties of the Al/Cu joints, tensile shear tests were performed at room temperature with a constant speed of 1 mm/min using a ZwickRoell Z020 universal testing machine (ZwickRoell, Ulm, Germany). To compensate for the asymmetry of the single overlap joint, an offset was set for the clamping device. Cross-sections of the welded Al/Cu joints were cut parallel to the OD, grounded, and polished for the microstructural characterization. Figure 1e shows two cut positions. The surface deformation of the upper joining partner, the fracture surface after the tensile shear test, and the prepared cross-section were analyzed using a Zeiss Smartzoom 5 digital optical microscope (OM) (Zeiss, Oberkochen, Germany). To observe further details of the microstructure, a Zeiss EVO 15 scanning electron microscope (SEM) (Zeiss, Oberkochen, Germany) with energy-dispersive X-ray spectroscopy (EDX) and a Zeiss Sigma with EDX and electron backscatter diffraction (EBSD) techniques were utilized for this study. The temperature evolution during the welding process was measured using a type K thermocouple, which was embedded as close as possible to the center of the weld zone at the weld interface, as shown in Figure 1d. To implant the thermocouple, a channel was sealed on the copper surface.

## 3. Results and Discussion

### 3.1. Plastic Deformation on the Al Surface

During USMW, the pyramid-profile tips of the sonotrode and anvil gradually penetrated into the joining partners as a result of the plastic deformation of the metals to be joined. Therefore, the indentation that appeared on the specimen surface varied with respect to the welding time. In this study, the analysis was focused on the plastic deformation of the upper specimen. Owing to the different mechanical performances, EN AW 1050 H24 more easily deforms than EN CW 008A R240. Furthermore, the oscillation amplitude decreased during the transmission from the sonotrode to the anvil, which also resulted in a larger deformation on the upper joining partner. The plastic deformation was characterized by two features: sonotrode indentation in the horizontal direction and base material compression in the vertical direction.

**Figure 2:**
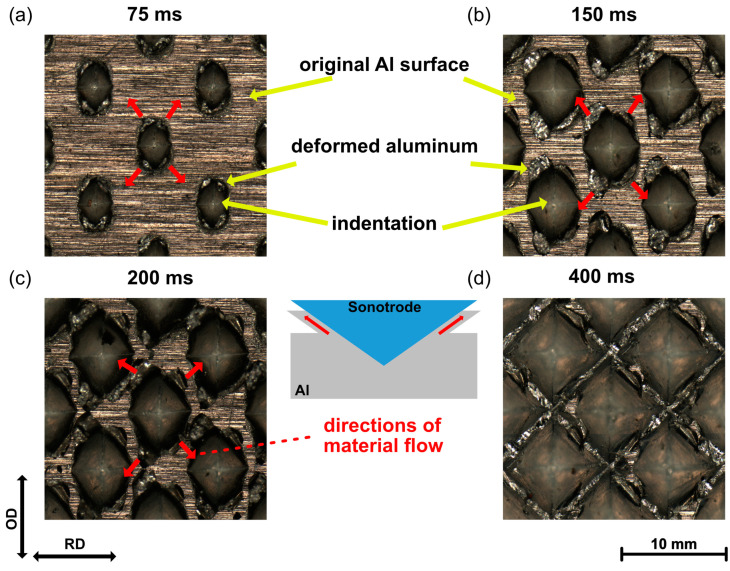
Indentation on the Al surface at different weld times: (**a**) 75 ms; (**b**) 150 ms; (**c**) 200 ms; (**d**) 400 ms, with u = 28 µm, and F_us_ = 700 N.

The appearance of the Al surface at different welding times was observed using a ZEISS Smartzoom 5 OM. Figure 2 shows the evolution of the indentation. At the initial phase of the welding process, the original Al surface with rolling marks was still observed, and the indentation appeared to be relatively small in rhombic shape. It can be seen that the plastic deformation started in the contact area under the sonotrode tip. As the welding process proceeded, the indentation gradually expanded, and the deformed Al flowed into the valley areas of the sonotrode tip. The directions of material flow are indicated by the red arrows in Figure 2. As can be seen, these directions tended to the OD because of the severe shear deformation. When the welding time reached 400 ms, the original Al surface was almost removed and the Al crests between indentation became sharp, which was fit to the outline of the sonotrode tips. Because there was no space for the deformed Al to flow in, no significant change was observed with further increases in welding time.

**Figure 3:**
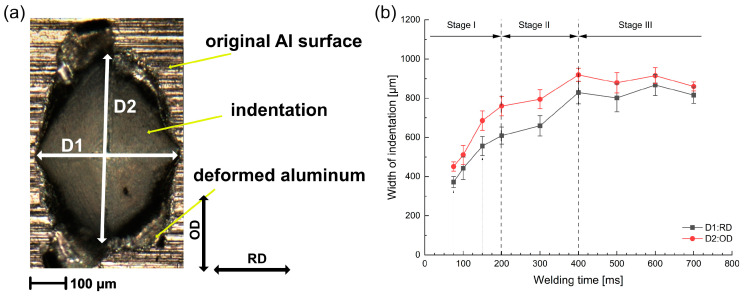
(**a**) Definition of widths for indentation D1 and D2. (**b**) Variation in widths of indentation D1 and D2 with welding time.

To quantitatively analyze the indentation on the Al surface, the widths of indentation D1 along the RD and D2 along the OD were measured, as illustrated in Figure 3a. The widths of the indentation are plotted against the welding time in Figure 3b. The evolution of indentation showed a similar tendency in both directions. However, the width D2 was constantly larger than D1 during USMW, which was caused by the tendency as a result of the vibration in the OD.

According to the increasing rate of the indentation, the ultrasonic welding process could be divided into three stages. In Stage I (<200 ms), the widths of the indentation rapidly grew in both directions. Moreover, the difference between D1 and D2 increased with increasing welding time and reached up to 150 µm at 200 ms. This was supposedly caused by the increase in the relative movement at the sonotrode/Al interface. In Stage II (200–400 ms), the increasing rate as well as the width difference between D1 and D2 decreased gradually. At the welding time of 400 ms, the width reached the maximum value of about 920 µm in the OD and 830 µm in the RD. During Stage III (>400 ms), the widths of the indentation remained nearly unchanged with some fluctuation, which indicated the complete penetration of the pyramid-profile tips.

**Figure 4:**
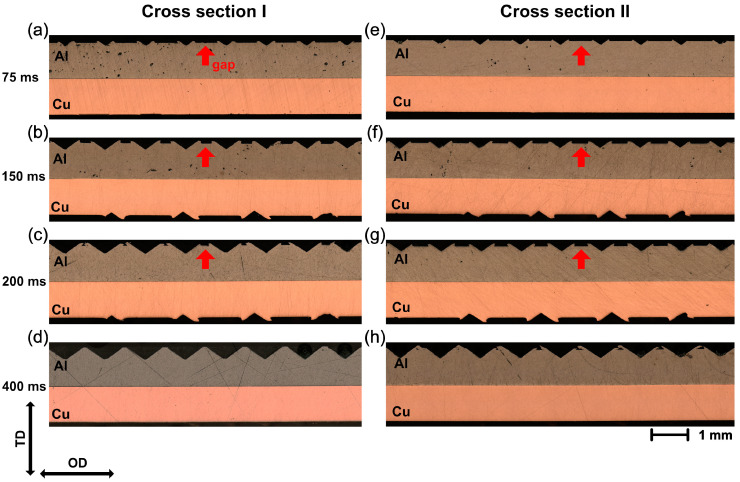
Optical micrographs of cross-sections for the Al/Cu joint at different welding time: (**a**–**d**) cross-section I; (**e**–**h**) cross-section II.

To evaluate the compaction behavior in the vertical direction, two cross-sections parallel to the OD were prepared. As shown in Figure 1e, cross-section I was close to the edge of the plastically deformed area, while cross-section II was in the middle of the weld zone. Figure 4 shows the optical micrographs of cross-sections I and II at different welding times. It can be noticed that the Al surface became more and more uneven in both cross-sections as the welding time increased. Owing to the stress concentration, a V-shaped valley formed at the contact area under the sonotrode tip. With additional shear stress caused by ultrasonic oscillation, the deformed Al was gradually driven into the free space between the sonotrode tip, resulting in a crest over the original Al surface. At short welding times, a gap between the two crests could be clearly obtained, as indicated by the red arrows in Figure 4. When the welding time reached 400 ms, the original Al surface was almost completely covered by the deformed Al and was not visible anymore.

**Figure 5:**
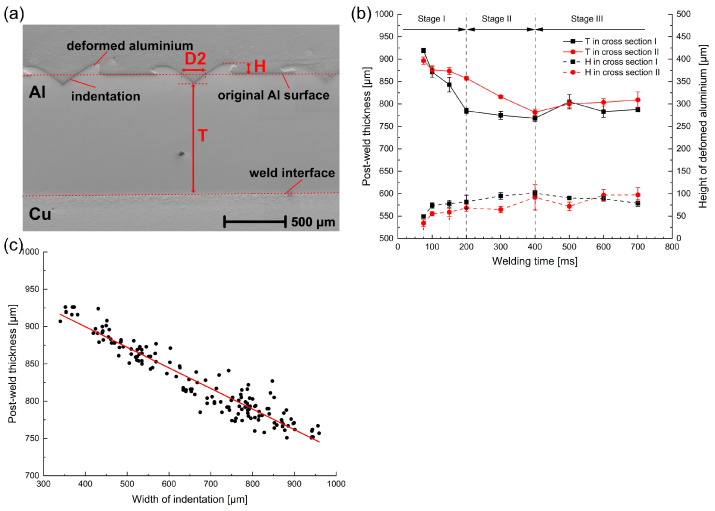
(**a**) Definition of postweld thickness T and height of deformed Al H, (**b**) variation in post-weld thickness T and height of deformed Al H with welding time, and (**c**) postweld thickness T vs. width for indentation D2.

To analyze the penetration process more accurately, the postweld thickness T [8] and the height of deformed Al H were measured, as defined in Figure 5a. The results are plotted in Figure 5b, where both cross-sections show a similar evolution of the plastic deformation on the Al surface during the welding process.

At the beginning of the welding process, the thickness of the Al decreased with a high compaction rate, and the difference between both cross-sections was relatively small. More than 55% of the total penetration depth was reached within 100 ms. Correspondingly, the height of the deformed Al increased up to 77 µm, nearly 80% of the maximum. As the welding process progressed, the penetration process of the sonotrode gradually slowed down and quickly reached a saturation condition. The final postweld thickness was about 770 µm, while the height of the deformed Al reached about 97 µm. However, the postweld thickness reached the maximum much faster in cross-section I than in cross-section II. This implied an inhomogeneous pressure distribution. A higher pressure was located at the edge of the weld zone, which could accelerate the formation process of the joints.

To identify the relationship between the deformation in the vertical and horizontal directions, the postweld thickness was correlated to the width of indentation, which is plotted in Figure 5c. It can be seen that the postweld thickness had a linear tendency to proportionally decrease with the width of indentation, which indicated that the specimen simultaneously deformed in the vertical and horizontal directions.

### 3.2. Microstructural Evolution at the Weld Interface

To comprehend the deformation behaviors and the microstructure evolution at the weld interface, the cross-sections from Section 3.1 were investigated using OM, SEM, EBSD, and EDX. As shown in Figure 4, a flat weld interface was observed at the weld interface, and the swirl-like structures reported by Zhao et al. [17] and Bakavos et al. [44] did not appear. Thus, no obvious mechanical interlocking was formed, which is associated with the significant hardness difference between Al and Cu [17,41].

**Figure 6:**
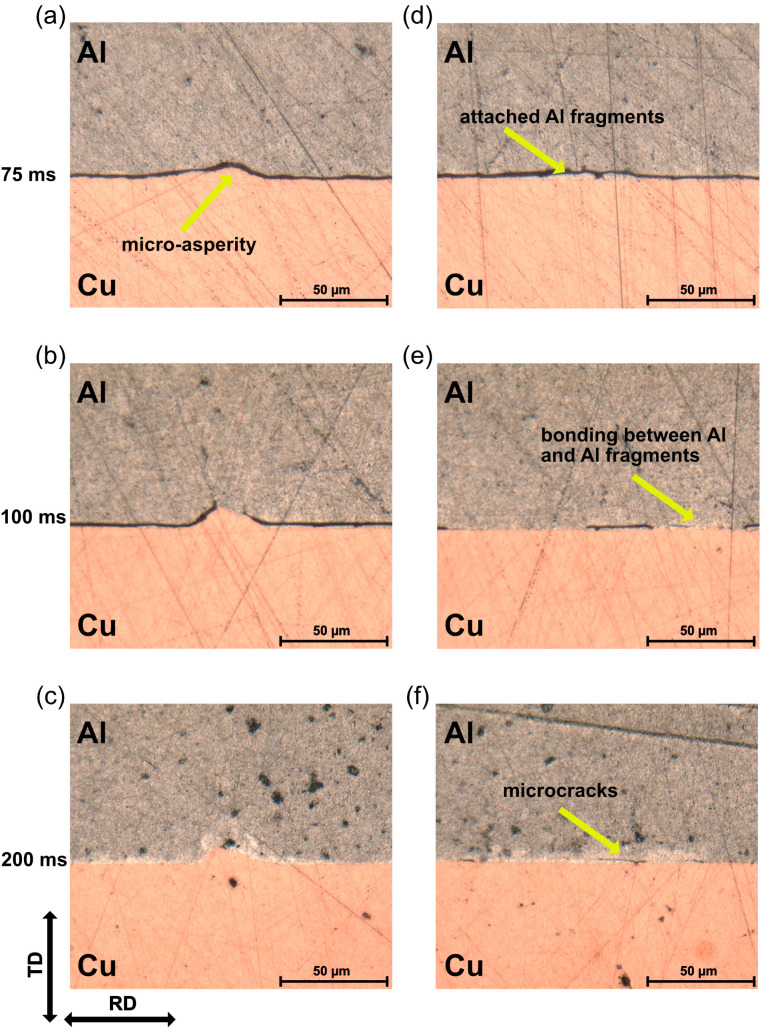
Optical micrographs of cross-sections at the weld interface: (**a**–**c**) microasperities; (**d**–**f**) locations with attached Al fragments.

The optical micrographs of cross-section I at different welding times in Stage I are shown in Figure 6. To learn the weld formation process, we focused on two characteristic regions: the microasperities (a–c) and the locations with attached Al fragments (d–f) on the Cu surface, which were mainly localized under the indentation of the sonotrode.

As shown in Figure 6a,d, a gap was located at the weld interface in both regions when the welding time was shorter than 75 ms. Nevertheless, the attached Al fragments were discovered on the copper surface in the unbonded regions, which indicated that a metallurgical bonding between Al and Cu was already achieved at this time. Furthermore, Figure 6d shows that the Al surface fits the outline of the Al fragments. It is suggested that the initial bonding between Al and Cu fractured on the Al side under the shear stress, resulting in the formation of attached Al fragments on the Cu surface.

This gap between Al and Cu was clearly reduced, and the microasperity of Cu penetrated into Al at a welding duration of 100 ms. As shown in Figure 6b, a strong bonding was formed on the top of the microasperity. Simultaneously, local bonding between the Al and Al fragments can be observed in Figure 6e, which was characterized by bonding with some inside cracks. Additionally, more and more unbonded regions on the Cu surface were covered by attached Al fragments, which could have promoted the formation of the bonding between the Al and Al fragments.

With increasing welding time to 200 ms, the gap at the weld interface was almost eliminated; the microwelds spread across the interface and coalesced with each other. As a result, a continuous weld line was observed at the weld interface. As shown in Figure 6c, the microasperity penetrated completely into the Al, where mechanical interlocking could be generated and enhance the joint strength. Nevertheless, there were still a few microcracks that appeared on the Al side, which further confirmed the bonding formed between the Al and attached Al fragments. These observations were in agreement with the results reported by Ma et al. [43].

**Figure 7:**
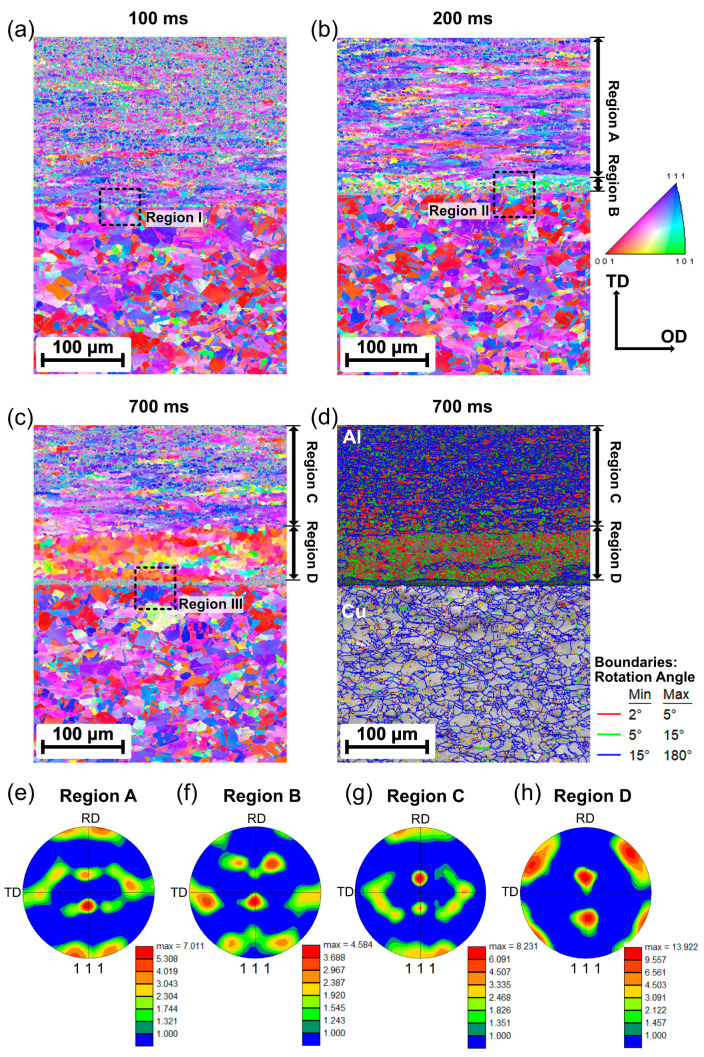
Inverse pole figures maps of the Al/Cu joint at different welding times: (**a**) 100 ms, (**b**) 200 ms, and (**c**) 700 ms. (**d**) Grain boundary character of (**c**); (**e**–**h**) {111} pole figure extracted from each region of the inverse pole figure maps.

To examine the evolution of the grain structure and texture, EBSD analyses were performed at the weld interface of cross-section II. The approximate location was near the indentation, where the plastic strain locally concentrates according to Chen et al. [45]. The inverse pole figure (IPF) maps of the Al/Cu joints welded at 100 ms, 200 ms, and 700 ms are presented in Figure 7a–c, respectively. The initial microstructures of aluminum- and copper-based materials were characterized by the elongated grains along the rolling plane due to the mechanical deformation during cold rolling.

It can be seen that during the welding process, the Al near the weld interface significantly deformed, while no obvious microstructure changes were observed on the Cu side near the weld interface. At a welding time of 100 ms, the Al side had roughly the same microstructure as the base material, as shown in Figure 7a. However, a continuous noisy area was found at the weld interface, where no representative information was found with EBSD. This phenomenon could be associated with the gaps or the small grain size, which was below the EBSD resolution. According to Fujii et al. [46], nanosized grains can be generated around the weld interface due to high-strain-rate shear deformation and can promote weld formation during USMW.

When the welding time reached 200 ms, two regions with clear microstructural differences were observed: Region A showed a similar microstructure with elongated grains as the base material, while Region B consisted of fine and equiaxed grains with changed orientation near the welding interface. This microstructure was formed by dynamic recrystallization (DRX) due to frictional heat and plastic deformation during USMW. Figure 7e,f show the {111} pole figures extracted from Regions A and B, respectively. In Region A, the typical rolling texture for face-centered cubic (FCC) metals was observed, which was similar to the Al-base material. In Region B, a {111} <110> texture was detected near the weld interface, which is known to be one of the ideal simple shear orientations [47]. This indicated that dislocations glided mainly along the FCC slip systems {111} <110> during the USMW process and were induced by shear deformation. Similar microstructures were also reported for the USMW of Al/Al joints [48,49] and Al/steel joints [50].

When the welding time increased from 200 to 700 ms, the thickness of the recrystallized regions elevated from approximately 30 µm up to 65 µm. As shown in Figure 7c, the gathering of coarse grains with almost the same orientation was observed in Region D, while Region C still showed the microstructure of the Al-base material. The grain size in Region D appeared to be larger than that of the base materials, which contributed to a low level of noise in the IPF map. Moreover, many subgrains enclosed by low-angle grain boundaries (LAGBs) were observed in Region D. In Figure 7d, the misorientation angle of LAGBs ranges from 2° to 5° for the red lines and 5° to 15° for the green lines. This microstructure was formed by the significant growth of the recrystallized grains due to the long welding time. The {111} pole figures extracted from Regions C and D are presented in Figure 7g,h. Region C shows a rolling texture similar to that of Region A and the Al-base material. In contrast, a {110} <001> Goss texture was observed in Region D, which was attributed to the annealing process [51,52].

**Figure 8:**
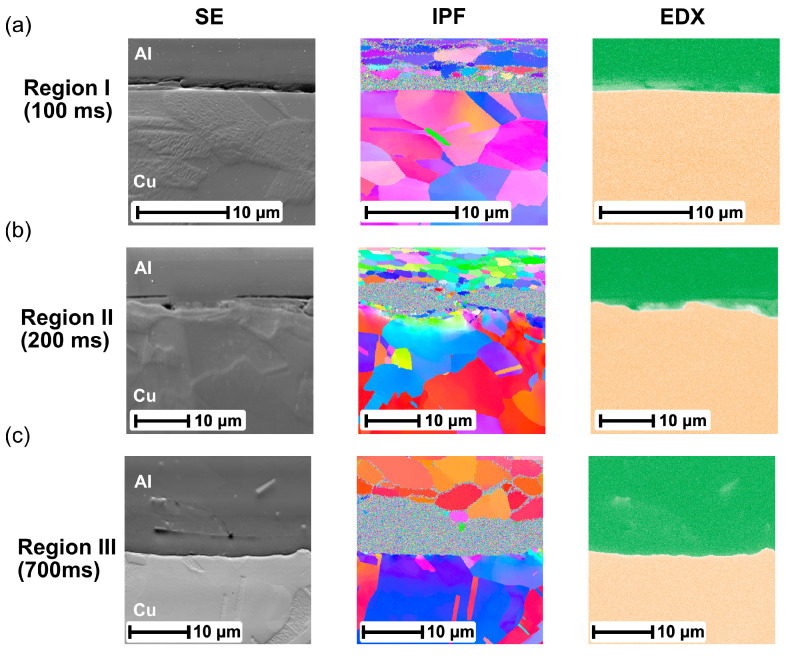
Magnified view of (**a**) region I at 100 ms, (**b**) region II at 200 ms, and (**c**) region III at 700 ms.

To investigate the deformation behavior at the weld interface more accurately, Regions I, II, and III, marked at the weld interface in Figure 7a–c, respectively, are magnified in Figure 8. SEM micrographs and the corresponding EDS analyses were also performed at this location. The IPF map shows that the thickness of the noisy area gradually increased up to 9 µm with increasing welding time. Furthermore, numerous gaps and cracks were found in this noisy area, as shown in the SEM images. The EDX mapping revealed that such gaps and cracks were fully located on the Al side. When combined with the OMs, it indicated that the rest of the materials in the noisy area corresponded to nanosized Al layers. The bonding between the Al and attached Al fragments could be formed in this region. However, no obvious IMC layer was generated at the weld interface even for a long welding time.

### 3.3. Temperature Evolution at the Weld Interface

**Figure 9:**
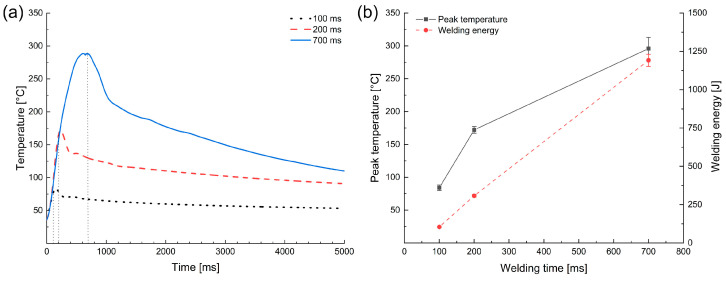
(**a**) Temperature–time profiles and (**b**) peak temperature and welding energy at different welding times.

To further verify the microstructural evolution during USMW, the interfacial temperature was measured by embedding a thermocouple at the center of the weld zone. The temperature profiles and the peak temperature during USMW are presented in Figure 9a,b, respectively. A similar increase in interface temperature was observed with increasing welding time. The temperature reached a maximum at the end of the welding process. Because of the excellent thermal conductivity of aluminum and copper, the temperature decreased rapidly after the welding process.

However, the temperature gradient decreased as the welding time increased. During USMW, the heat was generated and introduced into the weld area due to plastic deformation and interface friction [53]. At the initial phase of the welding process, the contact resistance between two joining partners was high, and most of the frictional work was performed at the weld interface. With the expansion of microwelds, more vibrating energy was delivered into the sonotrode/Al interface, and the temperature gradient of the weld area decreased. In contrast, a mostly linear relationship existed between the welding energy and welding time, as shown in Figure 9b.

It can be seen that the average peak temperature was only 84 °C at 100 ms and reached about 295 °C at 700 ms, which are far below the melting point of Al (660 °C). Additionally, the highest peak temperature was still much lower than the eutectic transition temperature (548.2 °C) in the Al–Cu equilibrium phase diagram [54]. This restricted the formation of the effective IMCs at the Al/Cu interface, which corresponded well to the results from the EDX analysis in Figure 8.

When compared with the results of EBSD, it should be noted that the interface temperature exceeded the recrystallization temperature (240 °C) at a welding time of approximate 500 ms. However, recrystallized grains were actually observed at a welding time of 200 ms. Due to the high-strain-rate deformation, a number of microstructure defects, such as vacancy and dislocation, were generated at the weld interface, which could have accelerated the diffusion phenomenon [50,55,56]. Thus, recrystallization can happen during USMW, even at low temperatures.

### 3.4. Mechanical Properties and Fractography Analysis

To evaluate the monotonic mechanical properties of the Al/Cu joints, tensile shear tests were performed. The mechanical performance could be identified based on the tensile shear failure load, load–displacement curves, and corresponding failure modes.

**Figure 10:**
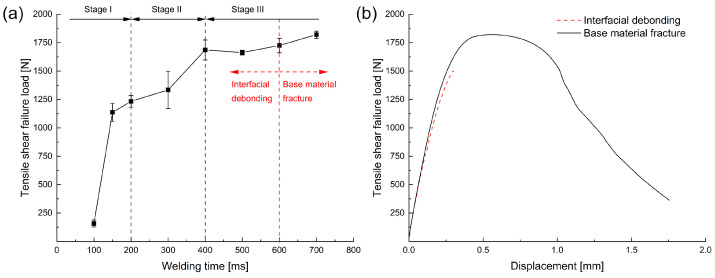
(**a**) Effect of welding time on the tensile shear failure load, and (**b**) load–displacement curves for interfacial debonding and base material fracture modes.

The tensile shear failure load of the joints as a function of welding time is presented in Figure 10a. When the welding time was 75 ms, there was no actual joint formed, and a few welded joints failed during the loading process with the preload (40 N). As the welding time increased from 100 to 400 ms, the failure load gradually increased. After then, the average failure load reached a plateau of 1663–1820 N, which was approximately 89–97% of that of the Al-base material. However, the fracture mode changed from interfacial debonding to base material fracture as the welding time exceeded 600 ms. Figure 10b shows the typical load–displacement behaviors for the two different failure modes. In the case of base material fracture, the load first increased up to the maximum, remained almost stable, and then decreased until the Al was completely separated into two pieces. In contrast, the interfacial debonding was characterized by purely elastic deformation and an abrupt separation between the two joining partners as the load increased.

**Figure 11:**
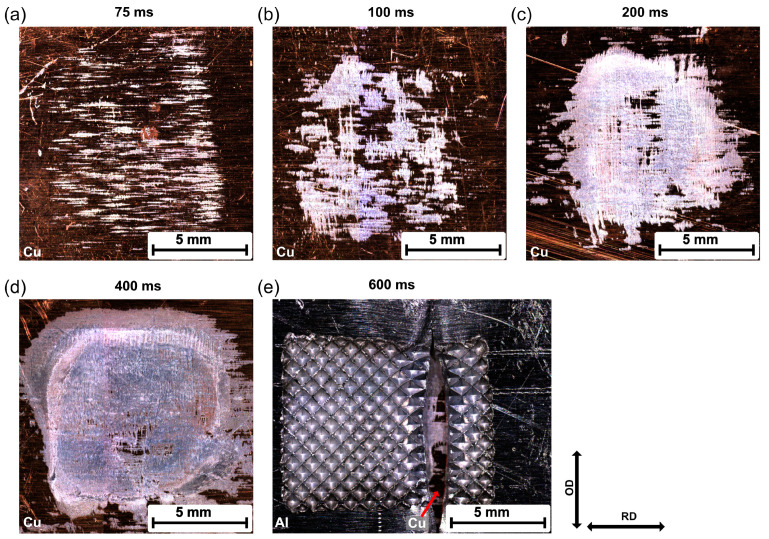
Fracture surfaces of Al–Cu joints after tensile shear tests: (**a**) 75 ms; (**b**) 100 ms; (**c**) 200 ms; (**d**) 400 ms; (**e**) 600 ms.

Figure 11 shows the representative fracture surfaces from the Cu side for the Al/Cu joints at different welding times. When the welding time was less than 600 ms, the failure passed through the welding interface, and some residual Al was observed on the Cu surface. However, this residual Al could be attributed to the generation of the attached Al fragment during USMW or fracture during the tensile shear test.

It can be seen that the morphology of the residual Al changed with increasing welding time. When the welding time was 75 ms, minimal residual Al remained on the Cu surface, and traces of Al oriented in line with the RD. Because no actual joint was formed at this time, this residual Al should have been the attached Al fragments shown in Figure 6. This suggests that the initial Al/Cu bonding mainly concentrated on the microasperities. As the welding duration increased, the microwelds grew in the OD, and large continuous welded areas were formed. However, the welded area propagated extremely inhomogeneously during the welding process, as can be seen in Figure 11b–d. Generally, most welded areas were located close to the edges of the weld zone, while some unbonded areas could still be observed at the center of the welding zone. This heterogeneous welded area could have been caused by the temperature, stress distribution, or surface morphology of the individual joining partners during USMW. When the welding time exceeded 600 ms, the bonding strength at the weld interface was stronger than the strength of the Al base material. Here, the failure crack propagated across the width of the aluminum, as illustrated in Figure 11e. Owing to the penetration of the sonotrode, extensive plastic deformation occurred at the aluminum surface and resulted in material thinning, which reduced the failure load of the Al base material.

To quantitatively analyze the fracture surface, a basic image processing techniques (IPT) was used and implemented in Python. The Python script was developed to measure the welded area by identifying the color code associated with each pixel and then marking the pixels within certain color codes as the welded area. Here, the green (G) value was used as the threshold value.

**Figure 12:**
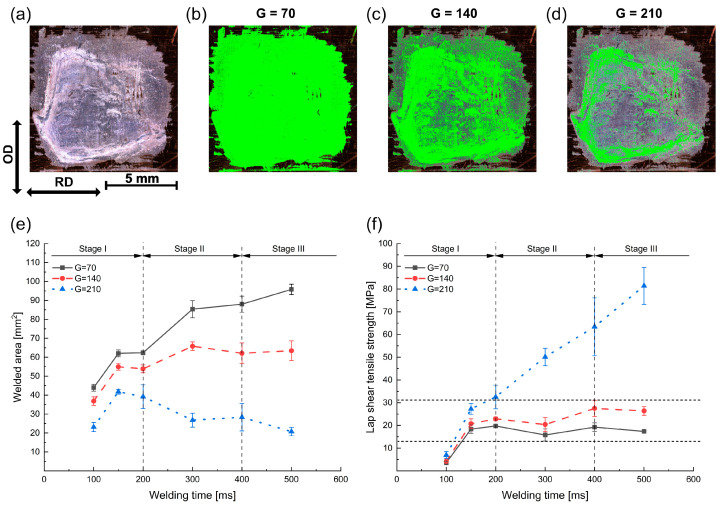
(**a**) Fracture surface of Al/Cu joints at 500 ms: the welded area as measured from the image processing technique (**b**) G = 70, (**c**) G = 140, and (**d**) G = 210. Variation in (**e**) welded area and (**f**) lap shear tensile strength with welding time.

An example of the fracture surface from an Al/Cu joint welded at 500 ms is shown in Figure 12a–d. The marked area reduced with an increase in the intensity of the G value from 70 to 210. The measured results for all fracture surfaces after tensile shear tests are summarized in Figure 12e. It can be seen that the welded area initially expanded with an increase in welding time and then remained almost stable in the time of 300 to 500 ms. Approximate 92% and 64% of the welding zone were identified as the real welded area for G = 70 and G = 140, respectively.

An interesting phenomenon was observed in case of G = 210. The welded area reached a maximum around 150–200 ms and then continuously decreased. As the welding process proceeded, the enhanced joint strength led to the increased thickness of the residual Al on the Cu surface after the tensile shear test. This caused a high gradient of the brightness contrast in OM images. As a result, more areas were marked as a “welded area” after a short welding time, although no strong bonding had formed in such regions.

Based on the tensile shear failure load (F_ts_) and the welded area (A_w_), the lap shear tensile strength (τ = F_ts_/A_w_) was calculated and is plotted in Figure 12f. It can be seen that the tensile strength remained nearly constant after 150 ms, which was closer to the limits of the Al base material. Because there was no significant improvement in the tensile strength, the increase in the failure load was likely the result of the expansion of the welded area, especially in the center of the weld zone. In contrast, the tensile strength reached a low point of approximately 5 MPa at a welding time of 100 ms, which suggested that most of the residual Al was the attached Al fragments.

**Figure 13:**
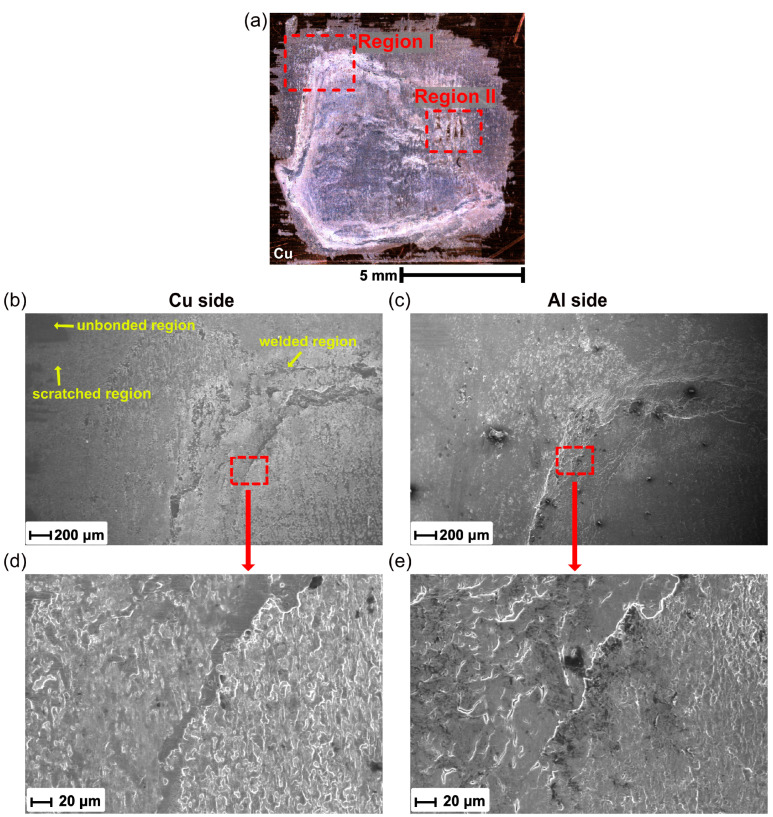
(**a**) Optical micrograph of fracture surface of Al/Cu joint, (**b**,**c**) SEM images of the fracture morphology for the Al/Cu joint in Region I, and (**d**,**e**) magnified view of selected region.

SEM was used to reveal more detail on the fracture surfaces, as shown in Figure 13a. Figure 13b,c show the higher magnification SEM images for Region I from the copper and aluminum sides, respectively. It can be seen that the fracture surface generally consisted of three different regions: unbonded region, scratched region, and welded region. In the unbonded region, some flat-looking type areas with the rolling marks of the base materials were observed. The scratched region mainly localized at the edges of the weld zone, and some attached aluminum debris was found here. Figure 13d,e show the magnified area of the box indicated in Figure 13b,c. Typical cleavage planes, shear ridges, and fracture dimples were observed in the welded region, revealing that a ductile–brittle hybrid fracture occurred in the tensile shear test. This may have been caused by the inhomogeneous temperature and stress distribution at the weld interface.

**Figure 14:**
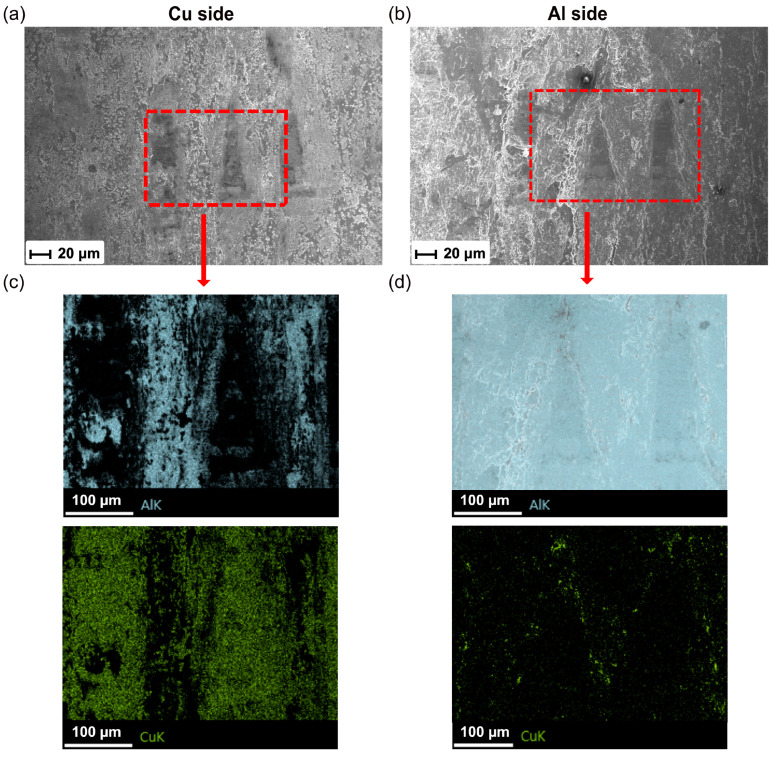
(**a**,**b**) SEM images of the fracture morphology for the Al/Cu joint in Region II, and (**c**,**d**) EDS maps of selected region.

Furthermore, Figure 14 shows the higher-magnification SEM images for Region II in Figure 13a. Few unbonded regions were clearly observed and were surrounded by fracture dimples. To characterize the chemical composition, the EDX analysis was performed and is presented in Figure 14c,d. It can be seen that the Cu side exhibited Al in the welded region, while no Al was detected in the unbonded region. In contrast, minimal Cu was found on the Al side and was located at the border between unbonded region and welded region, which suggested that plastic deformation also occurred on the Cu side at the weld interface during USMW.

### 3.5. Correlation between USMW Process and Mechanical Performance

In previous subsections, the plastic deformation and microstructural evolution during USMW were discussed. Both of them had a significant influence on the weld formation as well as the joint strength. The plastic deformation was induced by the welding force and the relative motion due to the ultrasonic oscillation. The results implied an inhomogeneous pressure distribution. Generally, higher pressure was located at the edge of the weld zone, which accelerated the formation process of joints. The examination of the fracture surfaces after the tensile shear test further confirmed that strong bonding formed initially at the edge and then expanded to the center, as illustrated in Figure 11. Based on the microstructural characterization, weld formation involved several substages: In the early stage, attached Al fragments were generated on the Cu surface thought the formation and fracture of the Al/Cu bonding. As the welding progressed, more and more unbonded regions on the Cu surface were covered by attached Al fragments. Simultaneously, metallurgical bonding formed between the Al and Al fragments. With further increases in welding time, microwelds spread across the weld interface, resulting in an increased welded area.

The mechanical properties of welded joints are strongly associated with the weld formation process. As illustrated in Figure 10, the failure load gradually increased until 400 ms and then remained almost stable. When combined with the plastic deformation and joint formation process, this change in the failure load could be deduced as follows: In Stage I (<200 ms), the strong bonding located mostly at the edge of the weld zone. The generation of attached Al fragments occurred mainly before 150 ms, which was characterized by the first increase in the welded area. However, the Al/Al fragments’ bonding gradually formed after 100 ms and led to a significant rise in the failure load. In Stage II (200–400 ms), the weld formation process mainly occurred at the center of the weld zone. Similar substages were observed: the generation of Al fragments from 200 ms to 300 ms and the formation of Al/Al fragments’ bonding after 300 ms. In Stage III (>400 ms), the weld area was completely formed with no more significant improvement in the failure load. Nevertheless, longer welding time was attributed to severe plastic deformation of the Al, which led to a change in the fracture mode from interfacial debonding to base material fracture.

## 4. Conclusions

In this study, the ultrasonic metal welding of Al/Cu joint was conducted for different welding durations. The plastic deformation of the joining partners and the microstructure evolution at the weld interface were qualitatively and quantitatively investigated. The main conclusions can be summarized as follows:

1. Owing to the welding force and ultrasonic oscillation, Al simultaneously deformed in the vertical and horizontal directions during USMW. After 400 ms, the sonotrode completely penetrated the upper joining partner, and the final postweld thickness was about 77% of the base material thickness. An inhomogeneous pressure distribution was observed, which affected the joint formation process and led to a heterogeneous distribution of the welded areas at the weld interface.
2. Microstructural characterization demonstrated that the joint formation process involves several substages: formation of Al/Cu bonding, generation of attached Al fragments, and formation of Al/Al fragment bonding. Additionally, the analysis of fracture surfaces showed that strong bonding was initially localized at the edge and then expanded to the center of the weld zone.
3. EBSD analysis showed that dynamic recrystallization occurred during USMW. The Al significantly deformed near the weld interface, and a recrystallized microstructure with a {111} <110> shear texture was formed at 200 ms. As the welding process proceeded, the recrystallized grains grew and showed a {110} <001> Goss texture near the weld interface.
4. The combined results from EBSD, SE and EDX revealed that a nanosized Al layer formed at the weld interface and confirmed the bonding process between Al and Al fragments. Furthermore, no obvious IMC layer was generated, even for a long welding duration.
5. The tensile shear failure load of joints gradually until 400 ms and then remained almost constant. The welded joint could achieve more than 80% of the mechanical performance of the base materials.

## Figures and Tables

**Table 1:** Selected mechanical properties of the base materials.

Sheet Materials	Yield Strength (MPa)	UTS (MPa)	Elongation at Fracture (%)	Hardness
EN AW 1050 H24	107.3 ± 5.4	129.0 ± 0.5	11.9 ± 0.7	39 HV 1
EN CW 008A R240	189.6 ± 2.4	252.1 ± 0.4	82.6 ± 5.5	81 HV 10

## Data Availability

The data presented in this study are available in the article.

## References

[1] Karden E., Shinn P., Bostock P., Cunningham J., Schoultz E., Kok D. (2005). Requirements for Future Automotive Batteries—A Snapshot. J. Power Sources.

[2] Granovskii M., Dincer I., Rosen M.A. (2006). Economic and Environmental Comparison of Conventional, Hybrid, Electric and Hydrogen Fuel Cell Vehicles. J. Power Sources.

[3] Maclean H.L., Lave L.B. (2000). Environmental Implications of Alternative-Fueled Automobiles: Air Quality and Greenhouse Gas Tradeoffs. Environ. Sci. Technol..

[4] Faria R., Moura P., Delgado J., De Almeida A.T. (2012). A Sustainability Assessment of Electric Vehicles as a Personal Mobility System. Energy Convers. Manag..

[5] Das A., Li D., Williams D., Greenwood D. (2018). Joining Technologies for Automotive Battery Systems Manufacturing. World Electr. Veh. J..

[6] Lee S.S., Kim T.H., Hu S.J., Cai W.W., Abell J.A. Joining Technologies for Automotive Lithium-Ion Battery Manufacturing: A Review. Proceedings of the International Manufacturing Science and Engineering Conference.

[7] Warner J. (2014). Lithium-Ion Battery Packs for EVs.

[8] Lee S.S., Hyung Kim T., Jack Hu S., Cai W., Abell J.A., Li J. (2013). Characterization of Joint Quality in Ultrasonic Welding of Battery Tabs. J. Manuf. Sci. Eng..

[9] Shin H.S., de Leon M. (2017). Mechanical Performance and Electrical Resistance of Ultrasonic Welded Multiple Cu-Al Layers. J. Mater. Process. Technol..

[10] Lee S.S., Kim T.H., Cai W., Abell J.A. (2014). Parasitic Vibration Attenuation in Ultrasonic Welding of Battery Tabs. Int. J. Adv. Manuf. Technol..

[11] Zwicker M.F.R., Moghadam M., Zhang W., Nielsen C.V. (2020). Automotive Battery Pack Manufacturing—A Review of Battery to Tab Joining. J. Adv. Join. Process..

[12] Das A., Li D., Williams D., Greenwood D. (2019). Weldability and Shear Strength Feasibility Study for Automotive Electric Vehicle Battery Tab Interconnects. J. Brazilian Soc. Mech. Sci. Eng..

[13] Brand M.J., Schmidt P.A., Zaeh M.F., Jossen A. (2015). Welding Techniques for Battery Cells and Resulting Electrical Contact Resistances. J. Energy Storage.

[14] Pati P.R., Das S., Satpathy M.P., Routara B.C., Sahoo S.K., Bhuyan S.K. (2020). Ultrasonic Spot Welding of Al-Cu Sheets: A Comprehensive Study. Mater. Today Proc..

[15] Cai W., Daehn G.S., Vivek A., Li J., Khan H., Mishra R.S., Komarasamy M. (2019). A State-of-the-Art Review on Solid-State Metal Joining. J. Manuf. Sci. Eng. Trans. ASME.

[16] Satpathy M.P., Mishra S.B., Sahoo S.K. (2018). Ultrasonic Spot Welding of Aluminum-Copper Dissimilar Metals: A Study on Joint Strength by Experimentation and Machine Learning Techniques. J. Manuf. Process..

[17] Zhao Y.Y., Li D., Zhang Y. (2013). Effect of Welding Energy on Interface Zone of Al–Cu Ultrasonic Welded Joint. Sci. Technol. Weld. Join..

[18] Liu J., Cao B., Yang J. (2017). Effects of Vibration Amplitude on Microstructure Evolution and Mechanical Strength of Ultrasonic Spot Welded Cu/Al Joints. Metals..

[19] Liu G., Hu X., Fu Y., Li Y. (2017). Microstructure and Mechanical Properties of Ultrasonic Welded Joint of 1060 Aluminum Alloy and T2 Pure Copper. Metals..

[20] Satpathy M.P., Mohapatra K.D., Sahoo S.K. (2018). Ultrasonic Spot Welding of Al–Cu Dissimilar Metals: A Study on Parametric Influence and Thermo-Mechanical Simulation. Int. J. Model. Simul..

[21] Satpathy M.P., Sahoo S.K. (2017). Mechanical Performance and Metallurgical Characterization of Ultrasonically Welded Dissimilar Joints. J. Manuf. Process..

[22] Elangovan S., Venkateshwaran S., Prakasan K. (2015). Experimental Investigations on Optimization of Ultrasonic Welding Parameters for Copper To Brass Joints Using Response Surface Method and Genetic Algorithm. Int. J. Adv. Eng. Res..

[23] Meng Y., Rajagopal M., Kuntumalla G., Toro R., Zhao H. (2020). Multi-Objective Optimization of Peel and Shear Strengths in Ultrasonic Metal Welding Using Machine Learning-Based Response Surface Methodology. Math. Biosci. Eng..

[24] Amiri N., Farrahi G.H., Kashyzadeh K.R., Chizari M. (2020). Applications of Ultrasonic Testing and Machine Learning Methods to Predict the Static & Fatigue Behavior of Spot-Welded Joints. J. Manuf. Process..

[25] Mongan P.G., Modi V., McLaughlin J.W., Hinchy E.P., O’Higgins R.M., O’Dowd N.P., McCarthy C.T. (2022). Multi-Objective Optimisation of Ultrasonically Welded Dissimilar Joints through Machine Learning. J. Intell. Manuf..

[26] Hyung Kim T., Yum J., Jack Hu S., Spicer J.P., Abell J.A. (2011). Process Robustness of Single Lap Ultrasonic Welding of Thin, Dissimilar Materials. CIRP Ann. Manuf. Technol..

[27] Das A., Masters I., Williams D. (2019). Process Robustness and Strength Analysis of Multi-Layered Dissimilar Joints Using Ultrasonic Metal Welding. Int. J. Adv. Manuf. Technol..

[28] Zhou B., Thouless M.D., Ward S.M. (2005). Determining Node-I Cohesive Parameters for Nugget Fracture in Ultrasonic Spot Welds. Int. J. Fract..

[29] Bao N., Zhao R. Design Optimization of Battery Holder for Electric Vehicle. Proceedings of the 2018 6th International Conference on Mechanical, Automotive and Materials Engineering (CMAME).

[30] Lee J.H., Lee H.M., Ahn S. (2003). Battery Dimensional Changes Occurring during Charge/Discharge Cycles—Thin Rectangular Lithium Ion and Polymer Cells. J. Power Sources.

[31] Kang B., Cai W., Tan C.A. (2014). Dynamic Stress Analysis of Battery Tabs Under Ultrasonic Welding. J. Manuf. Sci. Eng..

[32] Zhao N., Li W., Cai W., Abell J.A. (2014). A Fatigue Life Study of Ultrasonically Welded Lithium-Ion Battery Tab Joints Based on Electrical Resistance. J. Manuf. Sci. Eng..

[33] Ao S., Li C., Zhang W., Wu M., Dai Y., Chen Y., Luo Z. (2019). Microstructure Evolution and Mechanical Properties of Al/Cu Ultrasonic Spot Welded Joints during Thermal Processing. J. Manuf. Process..

[34] Braunovic M., Myshkin N.K., Konchits V.V. (2017). Electrical Contacts, Fundamentals, Applications and Technology.

[35] Lu Y., Song H., Taber G.A., Foster D.R., Daehn G.S., Zhang W. (2016). In-Situ Measurement of Relative Motion during Ultrasonic Spot Welding of Aluminum Alloy Using Photonic Doppler Velocimetry. J. Mater. Process. Technol..

[36] Sasaki T., Watanabe T., Hosokawa Y., Yanagisawa A. (2013). Analysis for Relative Motion in Ultrasonic Welding of Aluminium Sheet. Sci. Technol. Weld. Join..

[37] Chen K., Zhang Y., Wang H. (2017). Study of Plastic Deformation and Interface Friction Process for Ultrasonic Welding. Sci. Technol. Weld. Join..

[38] Lee S.S., Hyung Kim T., Jack Hu S., Cai W., Abell J.A. (2015). Analysis of Weld Formation in Multilayer Ultrasonic Metal Welding Using High-Speed Images. J. Manuf. Sci. Eng..

[39] Yang Y., Janaki Ram G.D., Stucker B.E. (2009). Bond Formation and Fiber Embedment during Ultrasonic Consolidation. J. Mater. Process. Technol..

[40] Wu X., Liu T., Cai W. (2015). Microstructure, Welding Mechanism, and Failure of Al/Cu Ultrasonic Welds. J. Manuf. Process..

[41] Balasundaram R., Patel V.K., Bhole S.D., Chen D. (2014). Effect of Zinc Interlayer on Ultrasonic Spot Welded Aluminum-to-Copper Joints. Mater. Sci. Eng. A.

[42] Fujii H.T., Endo H., Sato Y.S., Kokawa H. (2018). Interfacial Microstructure Evolution and Weld Formation during Ultrasonic Welding of Al Alloy to Cu. Mater. Charact..

[43] Ma Q., Song C., Zhou J., Zhang L., Ji H. (2021). Dynamic Weld Evolution during Ultrasonic Welding of Cu–Al Joints. Mater. Sci. Eng. A.

[44] Bakavos D., Prangnell P.B. (2010). Mechanisms of Joint and Microstructure Formation in High Power Ultrasonic Spot Welding 6111 Aluminium Automotive Sheet. Mater. Sci. Eng. A.

[45] Chen K., Zhang Y. (2015). Mechanical Analysis of Ultrasonic Welding Considering Knurl Pattern of Sonotrode Tip. Mater. Des..

[46] Fujii H.T., Shimizu S., Sato Y.S., Kokawa H. (2017). High-Strain-Rate Deformation in Ultrasonic Additive Manufacturing. Scr. Mater..

[47] Toth L.S., Gilormini P., Jonas J.J. (1988). Effect of Rate Sensitivity on the Stability of Torsion Textures. Acta Metall..

[48] Xie J., Zhu Y., Bian F., Liu C. (2017). Dynamic Recovery and Recrystallization Mechanisms during Ultrasonic Spot Welding of Al-Cu-Mg Alloy. Mater. Charact..

[49] Haddadi F., Tsivoulas D. (2016). Grain Structure, Texture and Mechanical Property Evolution of Automotive Aluminium Sheet during High Power Ultrasonic Welding. Mater. Charact..

[50] Fujii H.T., Goto Y., Sato Y.S., Kokawa H. (2016). Microstructure and Lap Shear Strength of the Weld Interface in Ultrasonic Welding of Al Alloy to Stainless Steel. Scr. Mater..

[51] Shimizu S., Fujii H.T., Sato Y.S., Kokawa H., Sriraman M.R., Babu S.S. (2014). Mechanism of Weld Formation during Very-High-Power Ultrasonic Additive Manufacturing of Al Alloy 6061. Acta Mater..

[52] Ji H., Wang J., Li M. (2014). Evolution of the Bulk Microstructure in 1100 Aluminum Builds Fabricated by Ultrasonic Metal Welding. J. Mater. Process. Technol..

[53] Chen K., Zhang Y. (2015). Numerical Analysis of Temperature Distribution during Ultrasonic Welding Process for Dissimilar Automotive Alloys. Sci. Technol. Weld. Join..

[54] Massalski T.B., Okamoto H. (1990). Binary Alloy Phase Diagrams.

[55] Gunduz I.E., Ando T., Shattuck E., Wong P.Y., Doumanidis C.C. (2005). Enhanced Diffusion and Phase Transformations during Ultrasonic Welding of Zinc and Aluminum. Scr. Mater..

[56] Samanta A., Xiao S., Shen N., Li J., Ding H. (2019). Atomistic Simulation of Diffusion Bonding of Dissimilar Materials Undergoing Ultrasonic Welding. Int. J. Adv. Manuf. Technol..

